# Modelling wave-induced sea ice break-up in the marginal ice zone

**DOI:** 10.1098/rspa.2017.0258

**Published:** 2017-10-04

**Authors:** F. Montiel, V. A. Squire

**Affiliations:** Department of Mathematics and Statistics, University of Otago, PO Box 56, Dunedin, New Zealand

**Keywords:** sea ice, ocean waves, marginal ice zone, wave scattering, fracture

## Abstract

A model of ice floe break-up under ocean wave forcing in the marginal ice zone (MIZ) is proposed to investigate how floe size distribution (FSD) evolves under repeated wave break-up events. A three-dimensional linear model of ocean wave scattering by a finite array of compliant circular ice floes is coupled to a flexural failure model, which breaks a floe into two floes provided the two-dimensional stress field satisfies a break-up criterion. A closed-feedback loop algorithm is devised, which (i) solves the wave-scattering problem for a given FSD under time-harmonic plane wave forcing, (ii) computes the stress field in all the floes, (iii) fractures the floes satisfying the break-up criterion, and (iv) generates an updated FSD, initializing the geometry for the next iteration of the loop. The FSD after 50 break-up events is unimodal and near normal, or bimodal, suggesting waves alone do not govern the power law observed in some field studies. Multiple scattering is found to enhance break-up for long waves and thin ice, but to reduce break-up for short waves and thick ice. A break-up front marches forward in the latter regime, as wave-induced fracture weakens the ice cover, allowing waves to travel deeper into the MIZ.

## 1. Introduction

The Arctic marginal ice zone (MIZ) that separates open ocean from the interior pack ice is experiencing rapid changes as a result of high-latitude climate change. During summer, for example, its extent relative to the total sea ice area is expanding [1], suggesting an increasing presence of thinner, loosely packed ice floes. Changes in environmental forcings, e.g. heat, winds and ocean waves, acting in partnership with positive feedback processes, are responsible for this transformation. Ocean waves, in particular, have been observed to break up the sea ice under flexural failure and, therefore, to contribute to the increasing extent of the MIZ, which is, in turn, more sensitive to summer melting because of the increased total perimeter of the ice floes created [2]. Correspondingly, sea ice loss increases open water extent and allows for more energetic swell to develop in the Arctic Basin [3,4], with the potential to fracture the sea ice further and cause additional melting. Although indirect observational evidence of this positive feedback mechanism was proposed by Kohout *et al.* [5] in the Antarctic MIZ, its impact on sea ice extent has not been quantified. Modelling the two-way coupling between the wave and sea ice systems on oceanic scales is needed to remedy this shortcoming.

The vast majority of modelling studies on ocean wave interactions with sea ice have attempted to quantify wave attenuation and directional spreading as a result of scattering [6–9] and dissipation [10,11] by the constituent ice floes, within the scope of linear water wave theory. These effects have recently been parametrized in spectral wave models, e.g. WAVEWATCH III^®^, to complement the description of physical processes influencing ocean wave propagation on a global scale and assess the role sea ice has on wave climate in the polar seas [12–16], acknowledging that the validity of such parametrizations is still the subject of much current research.

By contrast, very little is known about the impact of ocean waves on the break-up of sea ice floes in the MIZ. Observations have shown that floe size distribution (FSD), defined as the statistical distribution of floe sizes (e.g. mean caliper diameter or diameter of a circular floe with the same surface area) in the MIZ, satisfies a power law for floes with size larger than *O*(10–100 m) [17–19], while a regime shift often occurs for smaller floes. Although the latter regime has also been fitted to a power law, typically with a smaller power exponent than that fitted for large floes, uncertainty exists about whether scale invariance is a defining feature of the FSD. For instance, Herman [20] fitted a Pareto distribution to a collection of FSD datasets and was able to capture different scaling characteristics for small and large floe sizes.

It is unclear how waves contribute to the emergence of these observed power-law regimes, as flexural failure is not expected to occur below a critical floe size of order *O*(10 m) [21], which would suggest that the floe number distribution should decrease to zero as floes become small unless other break-up mechanisms are imposed. In this study, we address this question by modelling the break-up of an ice cover under a sustained wave event, with the goal of establishing the FSD emerging from wave forcing alone, i.e. isolated from wind, collisions and any other sources of sea ice break-up.

Very few models have attempted to describe the break-up of sea ice in the MIZ due to waves alone. Dumont *et al.* [22] were the first to propose a numerical model for the transport of ocean waves in the MIZ due to scattering by the constituent ice floes, in which a parametrization of ice floe break-up was included. In each cell of the discretized spatial domain, the FSD was described by a power law and parametrized by its minimum, mean and maximum floe size. At each time step, the FSD was updated according to a break-up criterion (discussed later) depending on wave amplitude in the cell and floes repeatedly fracturing in half with a prescribed probability, preserving the power-law distribution. Williams *et al.* [23,24] extended the work of Dumont *et al.* [22] by considering a more realistic break-up criterion, but used the same parametrization of the FSD. These authors focused their analysis on estimating the maximum distance from the ice edge where break-up can take place, which they define as the MIZ width, and did not examine the evolution of the power-law FSD during the break-up process. Implementation of two-way coupling between large-scale sea ice models, e.g. CICE or neXtSIM, and spectral wave models based on these modelling approaches is currently being investigated [25,26].

Other modelling studies have considered the evolution of the FSD in the framework of large-scale sea ice models. Horvat & Tziperman [27] and Zhang *et al.* [28] independently proposed a continuum transport equation for the FSD, extending a similar approach for ice thickness distribution used for describing ice thickness in large-scale sea ice models. The FSD is advected in time and space subject to a prescribed horizontal ice velocity field and a number of sources and sinks that describe the effects of thermodynamics (melting and freezing), lead opening, ridging and fragmentation, noting the latter phenomenon is parametrized in a highly simplified manner (uniformly redistributed floe sizes). Horvat and Tziperman provide the most advanced approach, by considering the joint floe size and thickness distribution and process-informed parametrizations of the source and sink terms, particularly wave-induced floe fracture, which accounts for the strain field generated by a wave spectrum. Also note that Herman [20] showed that the Pareto distribution used to fit observational FSD data (discussed earlier) emerges as a stable solution to a stochastic model of FSD evolution based on the generalized Lotka–Volterra equation.

Here, the three-dimensional phase-resolving scattering model of wave energy attenuation in the MIZ reported by Montiel *et al.* [9] is enhanced with a floe break-up model, allowing us to investigate the two-way wave–MIZ coupling in an idealized setting. The MIZ is constructed as an array of circular elastic floes with prescribed FSD and the forcing field is approximated by a monochromatic plane wave. The solution to the wave interaction problem provides a full description of (i) the wave field throughout the MIZ and (ii) the bending experienced by each floe. The latter information is used to derive a measure of elastic deformation in each floe which, if larger than a critical value, results in floe fracture. The post-break-up updated FSD is then fed back into the geometrical description of the MIZ, leading to a new solution of the wave interaction, which is in turn used to approximate ice floe break-up. Running this feedback loop simulation a sufficient number of times, we reach a steady-state FSD, which depends on the ice and wave parameters. The main goals of this investigation are to (i) study the evolution of the FSD towards its steady state under repeated wave break-up events, (ii) determine the effect of multiple scattering on the steady-state FSD, and (iii) examine the dependence of the FSD on the ice and wave parameters of this model.

A key novel feature of the flexural failure model proposed here is that it accounts for the two-dimensional stress field defined over the surface of each deformed floe. It is based on the two-dimensional Mohr–Coulomb (MC) stress criterion, which assesses mechanical failure from the combined level of tensile and compressive deformations at each point of the floe. The MC stress criterion has been used to estimate fracture in the elasto-brittle rheological sea ice model neXtSIM under horizontal deformations and wave-induced flexure [26]. The latter fracture model is a simplified one-dimensional version of the MC criterion used here, as wave-induced ice flexure is approximated using an elastic beam model of ice floe. This is in line with previous flexural failure models [22–24,27,29,30], in which deformation at each point of the beam is simply quantified by the curvature of its vertical displacement function. To our knowledge, the wave-induced break-up model of ice floes considered here is the first one to account for the additional spatial dimension.

We do not attempt to compare our model with experimental measurements in this investigation. Ice floe break-up by ocean waves in the MIZ has been reported via either *in situ*, e.g. [13,30–32], or remote-sensing, e.g. [12,33,34], observations. These papers describe qualitative or quantitative changes in the FSD after a large wave event breaks up the MIZ. It is unclear, however, which physical processes have contributed to the observed changes in the FSD, as it is not possible to isolate the effect of waves. For this reason, we focus our analysis on gaining a theoretical understanding of how ocean waves may influence sea ice break-up in the MIZ and the associated FSD.

## 2. Preliminaries

We consider a three-dimensional seawater domain with constant finite depth *h* and infinite horizontal extent. Cartesian coordinates **x**=(*x*,*y*,*z*) are used to locate points in the water domain, such that the planes *z*=0 and *z*=−*h* coincide with the unperturbed free surface and the flat impermeable seabed, respectively. The seawater is approximated as an inviscid and homogeneous incompressible fluid with density *ρ*_0_≈1025 kg m^−3^.

A finite array of *N*_f_ compliant sea ice floes is assumed to be freely floating at the equilibrium surface of the water domain. Each floe is circular with uniform thickness *D* and Archimedean draught *d*=(*ρ*/*ρ*_0_)*D*, where *ρ*≈922.5 kg m^−3^ is the density of sea ice. The radius of floe *i* is *a*_*i*_ and its centre has coordinates in the horizontal plane (*x*_*i*_,*y*_*i*_). The horizontal region of seawater covered by a floe is defined by

2.1 $$\Omega_{i}=\{(x,y)\in R^{2}:{\left(x-x_{i}\right)}^{2}+{\left(y-y_{i}\right)}^{2}\leq a_{i}^{2}\},$$

for any *i*∈*I*, where *I*={1,2,…,*N*_f_}. We further denote their union as *Ω*=*Ω*_1_∪*Ω*_2_∪⋯∪*Ω*_*N*_f__ and the horizontal region covered by a free surface as $\Omega_{0}=R^{2}\setminus \Omega$.

We consider time-harmonic perturbations in the water with prescribed radian frequency *ω*. Assuming the flow is irrotational, the velocity field in the water domain is expressed as (∇,∂_*z*_) Re{(*g*/ i*ω*)*ϕ*(**x**) *e*^− i*ωt*^}, where ∇=(∂_*x*_,∂_*y*_) and *g*≈9.81 m s^−1^ is the acceleration due to gravity. The complex-valued potential field *ϕ* then satisfies Laplace's equation in the water domain

2.2 $$\nabla^{2}\phi +\partial_{z}^{2}\phi =0\;\text{for}\;\text{x}\in (\Omega_{0}\times (-h,0))\cup (\Omega \times (-h,-d)).$$

The condition of no normal flow on the seabed yields, in addition, the Neumann boundary condition

2.3 $$\partial_{z}\phi =0\;\text{on }z=-h.$$

We assume that the perturbations in the water induce a flow characterized by a vertical displacement at the free surface that is small compared with the horizontal characteristic length of the flow. The linearized free surface boundary condition then takes the form

2.4 $$\partial_{z}\phi =\alpha \phi \;\text{on }z=0\;\text{for }(x,y)\in \Omega_{0},$$

where *α*=*ω*^2^/*g*.

We introduce a coupling between the vertical deformations experienced by each ice floe and the flow in the water domain. Horizontal motions of the floes are neglected, a valid assumption in the regime considered here [35]. We model the ice floe vertical deformations using the Kirchhoff–Love thin-elastic plate theory. This model is valid provided (i) ice floe diameters are large compared with the thickness and (ii) vertical deformations are small compared with the thickness. The boundary condition on the underside of the ice floes is then given by

2.5 $$(F\nabla^{4}+\rho_{0}g-\rho D\omega^{2})\partial_{z}\phi =\rho_{0}\omega^{2}\phi \;\text{on}\;z=-d\;\text{for }(x,y)\in \Omega ,$$

where *F*=*ED*^3^/12(1−*ν*^2^) is the flexural rigidity of the floe, which depends on the effective flexural modulus *E* [21] and Poisson's ratio *ν*. The values *E*≈6 GPa and *ν*≈0.3 are commonly used for sea ice.

The requirement that each floe *i*∈*I* has no horizontal motion is written as

2.6 $$\partial_{r_{i}}\phi =0\;\text{on }r_{i}=a_{i}\;\text{for }-d<z<0$$

and the free edge conditions of zero bending moment and zero vertical shear stress at the edge are, respectively,

2.7 $$\left[r_{i}^{2}\nabla_{r_{i},\theta_{i}}^{2}-(1-\nu )(r_{i}\partial_{r_{i}}+\partial_{\theta_{i}}^{2})]\partial_{z}\phi =0\;\text{on }(r_{i},z)=(a_{i},-d\right)$$

and

2.8 $$[r_{i}^{3}\partial_{r_{i}}\nabla_{r_{i},\theta_{i}}^{2}+(1-\nu )(r_{i}\partial_{r_{i}}-1)\partial_{\theta_{i}}^{2}]\partial_{z}\phi =0\;\text{on }(r_{i},z)=(a_{i},-d),$$

where (*r*_*i*_,*θ*_*i*_) are the local polar coordinates with origin at the centre of floe *i*, defined by $\left(x-x_{i},y-y_{i})=(r_{i}\cos\theta_{i},r_{i}\sin\theta_{i}\right)$. The operator ∇_*r*_*i*_,*θ*_*i*__=(∂_*r*_*i*__+1/*r*_*i*_,(1/*r*_*i*_)∂_*θ*_*i*__) has also been introduced.

An ambient flow in the water domain is prescribed with potential

2.9 $$\phi^{\mathrm{am}}(\text{x})=\psi_{0}(z)\int_{-\pi /2}^{\pi /2}A^{\mathrm{am}}(\tau )\;e^{\;ik_{0}(x\cos\tau +y\sin\tau )}\;d\tau ,$$

which satisfies (2.2)–(2.4) and forces a non-trivial solution to the boundary-value problem (2.2)–(2.8). Equation (2.9) defines the coherent superposition of plane waves travelling in the positive *x*-direction at the surface of an open ocean and with amplitudes depending continuously on the propagation angle *τ* with respect to the *x*-axis. The flow in the vertical direction is described by the function $\psi_{0}(z)=\cosh k_{0}(z+h)/\cosh k_{0}h$, where *k*_0_ denotes the wavenumber of travelling waves in the open ocean (defined later).

We seek a solution of the boundary-value problem (2.2)–(2.8) of the form *ϕ*=*ϕ*^am^+*ϕ*^S^, where *ϕ*^S^ is the potential of the scattered wave field due to the presence of the ice floes in response to the ambient wave potential *ϕ*^am^. In the far field, the scattered wave potential satisfies the Sommerfeld radiation condition

2.10 $$\sqrt{r}(\partial_{r}-\;ik)\phi^{S}\to 0\;\text{as }r\to \infty ,$$

where $r=\sqrt{x^{2}+y^{2}}$.

## 3. Wave-scattering model

### (a) Single floe scattering

We decompose the potential in the exterior open water region adjacent to any floe *i* ∈ *I* (i.e. for *r*_*i*_>*a*_*i*_) as $\phi \equiv \phi_{\mathrm{ext}}^{\left(i\right)}=\phi_{\mathrm{in}}^{\left(i\right)}+\phi_{\mathrm{sc}}^{\left(i\right)}$, where $\phi_{\mathrm{in}}^{\left(i\right)}$ is the local incident wave potential generated by sources away from the floe and $\phi_{\mathrm{sc}}^{\left(i\right)}$ is the scattered wave potential generated due to the presence of floe *i*. Standard cylindrical eigenfunction expansions are used to express these potentials (e.g. [36])

3.1*a* $$\phi_{\mathrm{in}}^{\left(i\right)}(\text{x})=\sum_{m=0}^{\infty }\psi_{m}(z)\sum_{n=-\infty }^{\infty }a_{m,n}^{\left(i\right)}J_{n}(k_{m}r_{i})\;e^{\;in\theta_{i}}\;\text{for }\text{x}\in \Omega_{0}\times (-h,0)$$

and

3.1*b* $$\phi_{\mathrm{sc}}^{\left(i\right)}(\text{x})=\sum_{m=0}^{\infty }\psi_{m}(z)\sum_{n=-\infty }^{\infty }b_{m,n}^{\left(i\right)}H_{n}(k_{m}r_{i})\;e^{\;in\theta_{i}}\;\text{for }\text{x}\in \Omega_{0}\times (-h,0).$$

The potential $\phi \equiv \phi_{\mathrm{int}}^{\left(i\right)}$ in the interior region to floe *i* is expanded as

3.1*c* $$\phi_{\mathrm{int}}^{\left(i\right)}(\text{x})=\sum_{m=-2}^{\infty }\zeta_{m}(z)\sum_{n=-\infty }^{\infty }c_{m,n}^{\left(i\right)}J_{n}(k_{m}r_{i})\;e^{\;in\theta_{i}}\;\text{for }\text{x}\in \Omega_{i}\times (-h,-d),$$

in which the summation over *m* starts at −2 to account for the contribution of two vertical wave modes not present in the open water region. In (3.1), *J*_*n*_ and *H*_*n*_ denote the Bessel and Hankel functions of the first kind of order *n*, respectively.

The eigenfunctions describing the fluid flow in the vertical direction in the open water and ice-covered regions are given by

$$\psi_{m}(z)=\frac{\cosh k_{m}(z+h)}{\cosh k_{m}h},\;m\geq 0\;\text{and }\;\zeta_{m}(z)=\frac{\cosh\kappa_{m}(z+h)}{\cosh\kappa_{m}(h-d)},\;m\geq -2,$$

respectively.

The quantities *k*_*m*_, *m*≥0, are solutions of the open water dispersion relation

3.2 ktanh⁡kh=α.

We denote by *k*_0_ the only positive real root of (3.2), and by *k*_1_,*k*_2_,*k*_3_,… the infinite number of purely imaginary roots with positive imaginary part ordered such that *Im*(*k*_*m*+1_)>*Im*(*k*_*m*_) for all *m*≥1. The real root *k*_0_ is the wavenumber of horizontally travelling wave modes at the free surface of the open water region, while the imaginary roots *k*_*m*_, *m*≥1, are associated with horizontally evanescent wave modes decaying exponentially faster from their source for increasing values of *m*.

The quantities *κ*_*m*_, *m*≥−2, introduced in (3.1c) are solutions of the ice-covered dispersion relation

3.3 $$(\beta \kappa^{4}+1-\alpha d)\kappa \tanh\kappa (h-d)=\alpha .$$

The scaled elastic constant *β*=*F*/*ρ*_0_*g* has been introduced in (3.3). We denote by *κ*_0_ the only positive real root of (3.3), which is the wavenumber associated with horizontally travelling wave modes at the water–ice interface. In addition, *κ*_1_,*κ*_2_,*κ*_3_,… designate the infinitely many imaginary roots with increasingly large imaginary parts associated with evanescent waves, and *κ*_−2_ and *κ*_−1_ denote the two remaining complex roots with positive imaginary part that are associated with damped travelling wave modes.

A relationship exists between the unknown coefficients $a_{m,n}^{\left(i\right)}$, $b_{m,n}^{\left(i\right)}$ and $c_{m,n}^{\left(i\right)}$ of the eigenfunction expansions given in (3.1), as a consequence of the boundary conditions prescribed on the surface *r*_*i*_=*a*_*i*_. In addition to the condition of no horizontal motions (2.6), continuity of fluid pressure and normal velocity is imposed at the interface between the open water and ice-covered regions, i.e.

3.4 $$\phi_{\mathrm{ext}}^{\left(i\right)}=\phi_{\mathrm{int}}^{\left(i\right)}\;\text{and}\;\partial_{r_{i}}\phi_{\mathrm{ext}}^{\left(i\right)}=\partial_{r_{i}}\phi_{\mathrm{int}}^{\left(i\right)}\;\text{on }r_{i}=a_{i}\;\text{for }-h<z<-d.$$

A numerical solution is obtained by approximating the series expansions in (3.1) as partial sums, such that *m*≤*M* and |*n*|≤*N*, where *M* and *N* are convergence parameters. We apply the eigenfunction matching method (EMM) proposed by Montiel *et al.* [35]. It provides the following matrix relations between the coefficients:

3.5 $${\text{b}}^{\left(i\right)}={\text{S}}_{\mathrm{ext}}^{\left(i\right)}{\text{a}}^{\left(i\right)}\;\text{and}\;{\text{c}}^{\left(i\right)}={\text{S}}_{\mathrm{int}}^{\left(i\right)}{\text{a}}^{\left(i\right)},$$

where **a**^(*i*)^, **b**^(*i*)^ and **c**^(*i*)^ are the column vectors containing the coefficients $a_{m,n}^{\left(i\right)}$, $b_{m,n}^{\left(i\right)}$ and $c_{m,n}^{\left(i\right)}$, respectively. The vectors **a**^(*i*)^ and **b**^(*i*)^ have dimension (*M*+1)(2*N*+1), while **c**^(*i*)^ has dimension (*M*+3)(2*N*+1). Therefore, the matrices **S**^(*i*)^_ext_ and **S**^(*i*)^_int_ have dimensions ((*M*+1)(2*N*+1))^2^ and (*M*+3)(2*N*+1)×(*M*+1)(2*N*+1), respectively, and are referred to as the exterior and interior diffraction transfer matrices (DTMs). The DTMs describe the scattering properties of each floe.

### (b) Multiple scattering

We use a self-consistent method to resolve wave interactions with the array of *N*_f_ ice floes, in which the incident field $\phi_{\mathrm{in}}^{\left(i\right)}$ on each floe *i* ∈ *I* is given by the coherent superposition of the prescribed ambient field and the field scattered by all the other floes. This is expressed as

3.6 $$\phi_{\mathrm{in}}^{\left(i\right)}=\phi^{\mathrm{am}}+\sum_{j\in I,j\neq i}\phi_{\mathrm{sc}}^{\left(j\right)}\;\text{for all }i\in I.$$

This system of *N*_f_ equations can be solved after (i) writing the truncated cylindrical eigenfunction expansion of *ϕ*^am^ in the local coordinate system associated with floe *i*, (ii) applying Graf's addition theorem [37] to express $\phi_{\mathrm{sc}}^{\left(j\right)}$ in the local coordinate system associated with floe *i*, and (iii) using the exterior DTM of floe *i* to express the expansion coefficients of $\phi_{\mathrm{sc}}^{\left(j\right)}$ in terms of those of $\phi_{\mathrm{in}}^{\left(i\right)}$. This procedure yields a coupled system for the coefficients $a_{m,n}^{\left(i\right)}$, which can be inverted directly [35,38,39] or solved iteratively [40,41]. The local scattered field coefficients $b_{m,n}^{\left(i\right)}$ and $c_{m,n}^{\left(i\right)}$ are then computed directly using (3.5).

Numerical issues discussed by Montiel *et al.* [42,9] arise when the number of floes becomes larger than *O*(100) and/or when the floes are closely spaced. As a remedy, Montiel *et al.* proposed an algorithm that combines the direct approach with a domain decomposition technique, referred to as the slab-clustering method (SCM), in order to resolve wave interactions with *O*(10^4^–10^5^) floes. We only give a brief summary of the key steps of the SCM here and the reader is referred to the original papers [42,9] for further details.

(i) We cluster the array of floes into *N*_*s*_ slab regions parallel to the *y*-axis, so that each slab *q* is bounded by *x*=*ξ*_*q*−1_ and *x*=*ξ*_*q*_, with *ξ*_*q*−1_<*ξ*_*q*_, and contains the centre of *N*^(*q*)^ floes. In each slab *q*, we apply the direct method summarized above to obtain the matrix mapping
  3.7 $${\text{a}}_{q}={\text{S}}_{q}{\text{f}}_{q}$$
  between the vectors **a**_*q*_ containing the coefficients of the locally incident field $\phi_{\mathrm{in}}^{\left(i\right)}$ on each floe *i*=1,…,*N*^(*q*)^ in slab *q*, and **f**_*q*_ containing the coefficients of the forcing field composed of the ambient field and the field scattered by adjacent slabs expressed in the local polar coordinates of each floe in slab *q*.
(ii) We decompose the potential at each interface *x*=*ξ*_*q*_, 0≤*q*≤*N*_*s*_, as $\phi =\phi_{q}^{+}+\phi_{q}^{-}$, where $\phi_{q}^{\pm }$ is a field propagating or decaying in the positive/negative *x*-direction. Neglecting the vertical evanescent wave modes associated with the imaginary roots of (3.2), we approximate these components as
  3.8 $$\phi_{q}^{\pm }(\text{x})\approx \psi_{0}(z)\int_{\Lambda }A_{q}^{\pm }(\chi )\;e^{\;ik_{0}(\pm (x-\xi_{q})\cos\chi +y\sin\chi )}\;d\chi .$$
  The validity of this approximation was confirmed by Montiel *et al.* [9]. We have introduced the unknown amplitude functions $A_{q}^{\pm }(\chi )$ and the directional parameter *χ*∈*Λ*, where *Λ* is the integration contour which extends into the complex plane. It is defined by $\Lambda =\Lambda_{i}^{-}\cup \Lambda_{r}\cup \Lambda_{i}^{+}$, where $\Lambda_{i}^{\pm }=\pm \pi /2\mp (0,\infty )$ and *Λ*_*r*_=[−*π*/2,*π*/2]. A value of *χ*∈*Λ*_*r*_ corresponds to a plane wave travelling at angle *χ* with respect to the *x*-axis, while $\chi \in \Lambda_{i}^{\pm }$ corresponds to an evanescent wave decaying exponentially with *x*. Such evanescent wave components are generated by wave sources of the form (3.1b) from floes present in the adjacent slabs.
(iii) The amplitude functions $A_{q-1}^{\pm }(\chi )$ and $A_{q}^{\pm }(\chi )$, respectively, defined on the left and right boundary of slab *q*, are related through the following integral scattering relationships:
  3.9*a* $$A_{q-1}^{-}(\chi )=\int_{\Lambda }(R_{q}^{-}(\chi :\tau )A_{q-1}^{+}(\tau )+T_{q}^{-}(\chi :\tau )A_{q}^{-}(\tau ))\;d\tau$$
  and
  3.9*b* $$A_{q}^{+}(\chi )=\int_{\Lambda }(T_{q}^{+}(\chi :\tau )A_{q-1}^{+}(\tau )+R_{q}^{+}(\chi :\tau )A_{q}^{-}(\tau ))\;d\tau ,$$
  where $R_{q}^{\pm }(\chi :\tau )$ and $T_{q}^{\pm }(\chi :\tau )$ are the so-called reflection and transmission kernels of slab *q*. Semi-analytical expressions for these kernels were derived in [9]. They are obtained by combining (3.7) with mappings between cylindrical wave fields and plane wave fields.
(iv) We approximate numerically the scattering relationships () by discretizing the truncated integration contour $\tilde{\Lambda }={\tilde{\Lambda }}_{i}^{-}\cup \Lambda_{r}\cup {\tilde{\Lambda }}_{i}^{+}$, where ${\tilde{\Lambda }}_{i}^{\pm }=\pm \pi /2\mp \;i(0,\delta )$ for some *δ*≥0, sampling the amplitude and kernel functions at *N*_*Λ*_ discrete *χ* and *τ* values, and integrating this equation numerically (composite trapezoidal rule). We then obtain the following matrix equations:
  3.10 $${\text{A}}_{q-1}^{-}={\text{R}}_{q}^{-}{\text{A}}_{q-1}^{+}+{\text{T}}_{q}^{-}{\text{A}}_{q}^{-}\;\text{and}\;{\text{A}}_{q}^{+}={\text{T}}_{q}^{+}{\text{A}}_{q-1}^{+}+{\text{R}}_{q}^{+}{\text{A}}_{q}^{-},$$
  where ${\text{A}}_{q}^{\pm }$ are column vectors containing the sampled values of $A_{q}^{\pm }(\chi )$ and ${\text{R}}_{q}^{\pm }$ and ${\text{T}}_{q}^{\pm }$ are square matrices containing sampled values of the reflection and transmission kernels and the quadrature weights.
(v) We solve the set of 2*N*_*s*_ matrix equations defined by (3.10) for the amplitude vectors ${\text{A}}_{q}^{\pm }$, *q*=1,…,*N*_*s*_, using the iterative *S*-matrix method of Ko & Sambles [43]. This requires initialization of the forcing amplitudes as
  3.11 $$A_{0}^{+}(\tau )=A^{\mathrm{am}}(\tau )\;e^{\;ik\xi_{0}\cos\tau }\;\text{and}\;A_{N_{s}}^{-}(\tau )=0.$$
(vi) The local scattered wave fields in each slab *q* are obtained after transforming the plane wave forcing fields $\phi_{q-1}^{+}$ and $\phi_{q}^{-}$ into cylindrical regular wave fields with amplitudes contained in **f**_*q*_ and successively applying (3.7) and (3.5).

Convergence of the numerical method described here depends on the truncation parameters *M* and *N* in approximating cylindrical series expansions (3.1), and *δ* and *N*_*Λ*_ in approximating the plane wave expansions (3.8). For the computations carried out in this study, we set *M*=0, *N*=15, *δ*=1.2 and *N*_*Λ*_=*O*(100) (depending on the wave frequency), allowing us to compute scattered wave coefficients with three-digit accuracy. The choice *M*=0 simply means that we are ignoring the evanescent vertical wave modes. A comprehensive convergence analysis is conducted in [9] to justify these values.

## 4. Floe break-up criterion

### (a) Mohr–Coulomb stress

The thin elastic plate model of an ice floe considered here, with its underlying assumption of plane stress, allows us to write the Cauchy stress and strain tensors at each point as

4.1 $$S(r_{i},\theta_{i},t)=\left(\begin{array}{cc} \sigma_{r} & \sigma_{r\theta }\\ \sigma_{r\theta } & \sigma_{\theta } \end{array}\right)\;\text{and}\;E(r_{i},\theta_{i},t)=\frac{D}{2}\left(\begin{array}{cc} \varepsilon_{r} & \varepsilon_{r\theta }\\ \varepsilon_{r\theta } & \varepsilon_{\theta } \end{array}\right),$$

respectively, for any floe *i*∈*I*. The tensorial components with subscript *r* and *θ* are the normal stresses/strains in the radial and azimuthal direction, respectively, while the component with subscript *rθ* denotes the shear stress/strain. We can express the components of the strain tensor as [44]

4.2 $$\varepsilon_{r}=\partial_{r_{i}}^{2}w^{\left(i\right)},\;\varepsilon_{\theta }=\frac{1}{r_{i}^{2}}\partial_{\theta_{i}}^{2}w^{\left(i\right)}+\frac{1}{r_{i}}\partial_{r_{i}}w^{\left(i\right)}\;\text{and}\;\varepsilon_{r\theta }=\frac{1}{r_{i}}\partial_{r\theta_{i}}^{2}w^{\left(i\right)}-\frac{1}{r_{i}^{2}}\partial_{\theta_{i}}w^{\left(i\right)}.$$

Note that a typographical error in the expression of the shear strain given in [44] has been corrected here. We have defined the vertical displacement *w*^(*i*)^≡*w*^(*i*)^(*r*_*i*_,*θ*_*i*_,*t*) of floe *i*, which can be related to the potential on the undersurface of the floe through the kinematic condition

4.3 w(i)=Re(1α∂zϕint(i) e− iωt)on z=−dfor ri≤ai.

At each point of this undersurface and at a given time *t*, we then compute the components of the strain tensor from (4.2) after using (4.3) and (3.1*c*) to express the vertical displacement. Note that asymptotic formulae must be used for *ε*_*θ*_ and *ε*_*rθ*_ in the limit *r*_*i*_→0 [45].

The components of the stress tensor S are related to those of the strain tensor E through Hooke's law

4.4 $$\left(\begin{array}{c} \sigma_{r}\\ \sigma_{\theta }\\ \sigma_{r\theta } \end{array}\right)=\frac{ED}{2(1-\nu^{2})}\left(\begin{array}{ccc} 1 & \nu & 0\\ \nu & 1 & 0\\ 0 & 0 & 1-\nu \end{array}\right)\left(\begin{array}{c} \varepsilon_{r}\\ \varepsilon_{\theta }\\ \varepsilon_{r\theta } \end{array}\right).$$

We can diagonalize the stress tensor as $S=\tilde{\text{V}}D{\tilde{\text{V}}}^{T}$, where

4.5 $$\tilde{\text{V}}=[{\tilde{\text{v}}}_{1},{\tilde{\text{v}}}_{2}]\;\text{and}\;D=\mathrm{diag}\{\sigma_{1},\sigma_{2}\}.$$

The eigenvalues *σ*_1_≡*σ*_1_(*r*_*i*_,*θ*_*i*_,*t*) and *σ*_2_≡*σ*_2_(*r*_*i*_,*θ*_*i*_,*t*) of S are referred to as the principal stresses. The corresponding normalized eigenvectors ${\tilde{\text{v}}}_{1}(r_{i},\theta_{i},t)$ and ${\tilde{\text{v}}}_{2}(r_{i},\theta_{i},t)$ are orthogonal and define the so-called principal directions for which the shear stress vanishes in the polar coordinate frame centred at (*r*_*i*_,*θ*_*i*_). In the Cartesian frame with origin at the centre of floe *i*, the matrix of eigenvectors becomes

4.6 $$\text{V}(r_{i},\theta_{i},t)=\left(\begin{array}{cc} \cos\theta_{i} & -\sin\theta_{i}\\ \sin\theta_{i} & \cos\theta_{i} \end{array}\right)\tilde{\text{V}}(r_{i},\theta_{i},t).$$

We now derive a criterion for the break-up of an ice floe which incorporates some distinctive mechanical properties of sea ice. There exists a range of models for the mechanical failure of a large variety of materials under different types of loads ([46], ch. 2). They take the form $F(\sigma_{1},\sigma_{2})\geq 0$, where F=0 denotes a curve in the (*σ*_1_,*σ*_2_)-plane that corresponds to the onset of mechanical failure, and is often referred to as the yield curve. Here, we associate the ‘yielding’ of an ice floe with its fracture, which is reasonable for sea ice experiencing strain rates associated with wave periods of 5–20 s where plastic yield is negligible [47].

To our knowledge, no study has attempted to determine an appropriate model of wave-induced thin plate failure for sea ice, among the range of existing models in the literature of fracture mechanics. For this reason, we choose the simplest model of mechanical failure, applicable to both fracture and yield, and used for materials that exhibit significantly different values of tensile and compression strengths, defined as the maximum tensile and compression stresses that sea ice can experience before fracturing, respectively. In the case of sea ice, the tensile strength *σ*_*t*_ is typically an order of magnitude lower than the compression strength *σ*_*c*_ [48].

The failure model we use here is referred to as the Mohr–Coulomb (MC) criterion [46]. The yield curve is defined by

4.7 $$F(\sigma_{1},\sigma_{2})\equiv \sigma^{\left(\max\right)}(\sigma_{1},\sigma_{2})-\sigma^{\mathrm{MC}}=0,$$

where

4.8 $$\sigma^{\left(\max\right)}(\sigma_{1},\sigma_{2})=\max\{|\sigma_{1}-\sigma_{2}|+K(\sigma_{1}+\sigma_{2}),|\sigma_{1}|+K\sigma_{1},|\sigma_{2}|+K\sigma_{2}\},$$

with *K*=(*σ*_*c*_−*σ*_*t*_)/(*σ*_*c*_+*σ*_*t*_) and

4.9 $$\sigma^{\mathrm{MC}}=\frac{2\sigma_{c}\sigma_{t}}{\sigma_{c}+\sigma_{t}}.$$

**Figure 1.**
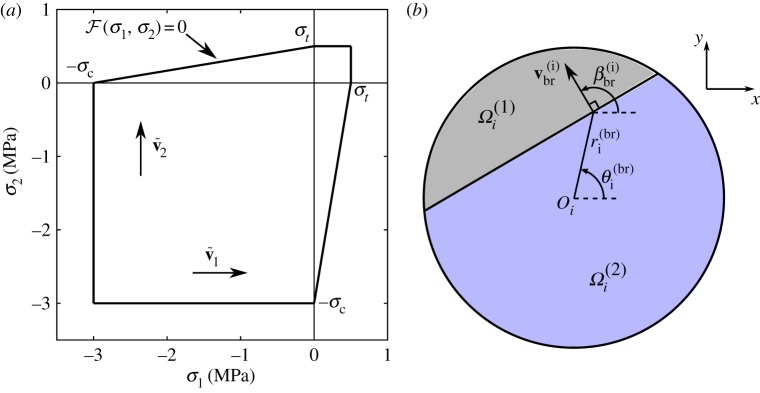
(*a*) MC yield curve $F(\sigma_{1},\sigma_{2})=0$ for a sea ice thin plate with tensile strength *σ*_*t*_= 0.5 MPa and compressive strength *σ*_*t*_=3 MPa. The principal directions ${\tilde{\text{v}}}_{1}$ and ${\tilde{\text{v}}}_{2}$ are also indicated. (*b*) Partition of the surface of floe *i* into two regions $\Omega_{i}^{\left(1\right)}$ and $\Omega_{i}^{\left(2\right)}$, resulting from the MC failure criterion (4.10) being satisfied. (Online version in colour.)

We refer to *σ*^(max)^(*r*_*i*_,*θ*_*i*_,*t*)≡*σ*^(max)^(*σ*_1_(*r*_*i*_,*θ*_*i*_,*t*),*σ*_2_(*r*_*i*_,*θ*_*i*_,*t*)) as the MC stress at the point (*r*_*i*_,*θ*_*i*_) and time *t*, and *σ*^MC^ as the MC critical stress. The yield curve F=0 is depicted in figure 1*a*. Neglecting the effect of fatigue, F is assumed to be stationary.

We now define the criterion for floe break-up as follows: a floe *i*∈*I* fractures into two smaller floes if the condition

4.10 $$\sigma_{\mathrm{br}}^{\left(i\right)}\equiv \max\left\{\sigma^{\left(\max\right)}(r_{i},\theta_{i},t),\;\text{for }(r_{i},\theta_{i},t)\in [0,a_{i})\times [0,2\pi )\times \left[0,\frac{2\pi }{\omega }\right)\right\}\geq \sigma^{\mathrm{MC}}$$

is satisfied. We refer to *σ*^(*i*)^_br_ as the potential break-up stress of floe *i*. The break-up criterion depends on the compressive and tensile strength of sea ice, which we estimate to be *σ*_*c*_=3 MPa and *σ*_*t*_=0.5 MPa, respectively. These values were chosen from empirical formulae reported by Timco & Weeks [48], assuming a brine volume fraction of approximately 0.04 and a strain rate of 2×10^−5^ s^−1^ consistent with a loading at a mid-range wave period of 10 s. These values are chosen to be realistic for sea ice in the MIZ, as opposed to attempting to replicate a particular observed field condition, which is likely to be associated with much variability for these parameters. The sensitivity of the break-up simulations conducted here with respect to the sea ice mechanical properties is not within the scope of the present work. It is noted that flexural strength of sea ice may be a better approximation for *σ*_*t*_ than the tensile strength used here for flexurally induced fracture. The value calculated from the empirical formula given in [48], however, is close to that for the tensile strength, so the distinction is actually of no practical importance.

### (b) Potential break-up stress in a single floe

We devise a sensitivity test to assess the potential for break-up of a single ice floe (i.e. *N*_*s*_=1 and *N*^(1)^=1) for a range of radii and wave periods. We prescribe a unidirectional plane wave with unit amplitude travelling in the positive *x* direction, by setting *A*^am^(*τ*)=*δ*(*τ*), where *δ* denotes the Dirac delta. We fix the water depth to *h*=200 m. We assume, without loss of generality, that the floe has its centre coinciding with the origin (*x*,*y*)=(0,0) of the horizontal Cartesian coordinate system. We compute the potential break-up stress *σ*_br_ for wave periods *T*=5–20 s, floe radii *a*=5–500 m and floe thicknesses *D*=1, 2 and 4 m.

**Figure 2.**
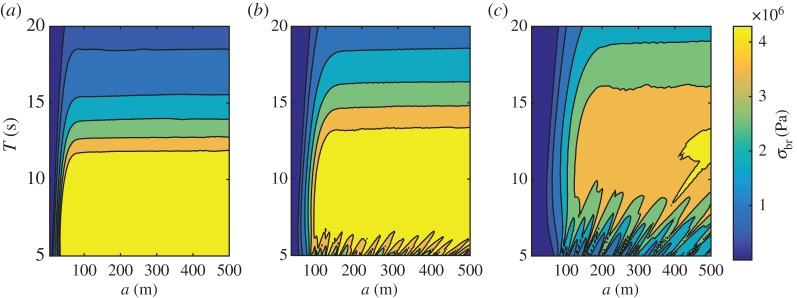
Filled contour plots of the potential break-up stress *σ*_br_ against wave period *T* and floe radius *a*, under unit amplitude plane wave forcing and for a single floe of thickness (*a*) *D*=1 m, (*b*) *D*=2 m and (*c*) *D*=4 m.

Filled contour plots of *σ*_br_ against wave periods and floe radii are shown in figure 2*a*–*c* for *D*=1, 2 and 4 m, respectively. The six contours displayed in each plot correspond to values of the potential break-up stress *σ*_br_=*eσ*^MC^ for *e*=0.5,1,2,3,4 and 5, noting that *σ*^MC^≈0.86 MPa.

For all three thicknesses considered, we generally observe that the potential break-up stress increases rapidly with the floe size before plateauing for floe radii greater than a certain value. This behaviour is seen at all wave periods for *D*=1 m and for periods greater than approximately 6 and 9 s for *D*=2 and 4 m, respectively. For shorter waves, *σ*_br_ oscillates with respect to *a*, suggesting resonances periodically induce large stress values, as the floe size approximately equals an integer multiple of the wavelength. We expect that similar oscillations would be observed for *D*=1 m at wave periods less than 5 s.

**Figure 3.**
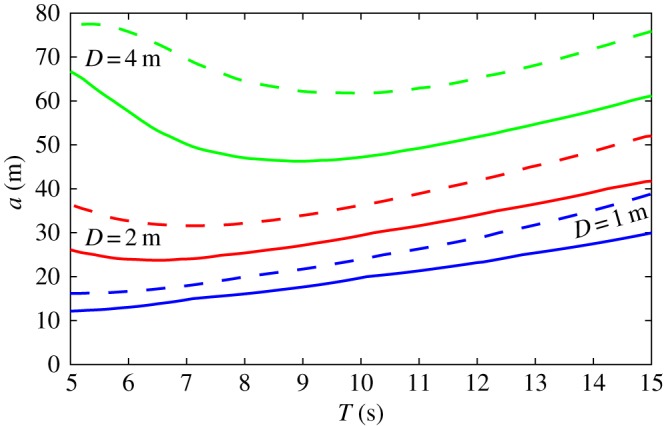
Variationof the MC radius *a*^MC^ (dashed lines) and the critical break-up radius *a*_crit_ (solid lines) with respect to *T* for floe thicknesses *D*=1 m (blue lines), *D*=2 m (red lines) and *D*=4 m (green lines). (Online version in colour.)

According to our break-up criterion (4.10), fracture occurs for *e*≥1, corresponding to the second contour in figure 2, i.e. the one separating the second and third darkest shades of blue. For all wave periods, it is seen that break-up occurs for all floe radii larger than a critical radius denoted by *a*^MC^ and referred to as the MC radius. It is shown as a function of wave period in figure 3 for the three ice thicknesses considered (dashed lines). We observe that it reaches a minimum at *T*=7 and 10 s for *D*=2 and 4 m, respectively, while it seems to approach a minimum near *T*=5 s for *D*=1 m. These correspond to resonant frequencies, when the floe diameter approximately coincides with the open water wavelength and half the ice-covered wavelength.

In figure 3, we further indicate by solid lines the radius corresponding to the contour defined by *e*=0.5 in figure 2. We denote it by *a*_crit_ and refer to it as the critical break-up radius. Although break-up does not occur for these radii in the single floe scattering simulations conducted here with a unit amplitude plane incident wave, numerical experiments showed that multiple interacting floes may generate more energetic wave forcing with the ability to fracture smaller floes as a result of constructive interference. In all subsequent simulations, we set *a*_crit_=*a*_crit_(*T*) as the minimum floe radius below which break-up cannot occur, unless otherwise discussed. This approximation allows us to perform multiple scattering simulations at a manageable computational cost. It should be noted that our arbitrary choice of *a*_crit_ is conservative in most cases in the sense that the probability that a floe with *a*<*a*_crit_ will fracture is small compared with the probability that a floe with *a*>*a*_crit_ does not.

## 5. Break-up model

We now seek to model the break-up of an array of ice floes, with the goal of determining the evolution of the FSD towards a steady state under repeated wave action and break-up events. To do this, we propose a numerical procedure that simulates the repeated break-up of an ice cover initially composed of *N*_f_ identical large floes with radius *a*_max_. To reduce the number of single floe solutions that need to be computed, we consider a finite number *N*_*r*_ of floe radii *a*^(1)^,…,*a*^(*N*_*r*_)^, such that *a*^(1)^=5 m and *a*^(*N*_*r*_)^=*a*_max_. We create a row vector *V*^(FSD)^_0_ of length *N*_*r*_, such that the *l*th entry contains the number of floes in the array with radius *a*^(*l*)^, for *l*=1,…,*N*_*r*_. For the initial configuration of floes, we then have

5.1 $$V_{0}^{\left(\mathrm{FSD}\right)}=(0,\ldots ,0,N_{f}).$$

The algorithm used for our break-up simulations is outlined as follows.

(i) Compute the exterior and interior DTMs associated with each floe radius *a*^(*p*)^, for *p*=1,…,*N*_*r*_, using the method discussed in §3a, and store them (the size of each DTM is *O*(10)).
(ii) Compute the solution of the multiple scattering problem using the method presented in §3b for the initial configuration of ice floes.
(iii) Compute the potential break-up stress *σ*^(*i*)^_br_ for each floe *i*∈*I* directly from the definition given in (4.10). In addition, define the normal break-up direction **v**^(*i*)^_br_ as the vector normal to the yield curve in the local Cartesian coordinate system of floe *i*, that is,
  5.2 $${\text{v}}_{\mathrm{br}}^{\left(i\right)}=\left(\begin{array}{c} \cos\beta_{\mathrm{br}}^{\left(i\right)}\\ \sin\beta_{\mathrm{br}}^{\left(i\right)} \end{array}\right)=\text{V}(r_{i}^{\left(\mathrm{br}\right)},\theta_{i}^{\left(\mathrm{br}\right)},t_{i}^{\left(\mathrm{br}\right)})\frac{\nabla_{\sigma }F}{|\nabla_{\sigma }F|},$$
  where $t_{i}^{\left(\mathrm{br}\right)}$ and $\left(r_{i}^{\left(\mathrm{br}\right)},\theta_{i}^{\left(\mathrm{br}\right)}\right)$ are the time and polar coordinates of the point of floe *i*, respectively, at which *σ*^(*i*)^_br_ is computed, and ∇_*σ*_=(∂_*σ*_1__,∂_*σ*_2__)^*T*^.
(iv) For each floe *i*, test the break-up criterion (4.10). If the inequality is not satisfied, floe *i* does not fracture and therefore remains in the array. If the inequality holds, however, remove floe *i* with radius *a*_*i*_ from the array and substitute it with two floes of radii $a_{i}^{\left(1\right)}<a_{i}$ and $a_{i}^{\left(2\right)}<a_{i}$ defined as follows: at the point $\left(r_{i}^{\left(\mathrm{br}\right)},\theta_{i}^{\left(\mathrm{br}\right)}\right)$, draw a straight line perpendicular to the vector **v**^(*i*)^_br_, partitioning the region *Ω*_*i*_ into two regions $\Omega_{i}^{\left(1\right)}$ and $\Omega_{i}^{\left(2\right)}$ as shown in figure 1*b*. We then define $a_{i}^{\left(1\right)}$ and $a_{i}^{\left(2\right)}$ as the radii of the discs with the area of regions $\Omega_{i}^{\left(1\right)}$ and $\Omega_{i}^{\left(2\right)}$, respectively. Their expressions are
  5.3 $$a_{i}^{\left(1\right)}=a_{i}\sqrt{\frac{\pi -2\theta_{0}-\sin2\theta_{0}}{2\pi }}\;\text{and}\;a_{i}^{\left(2\right)}=a_{i}\sqrt{\frac{\pi +2\theta_{0}+\sin2\theta_{0}}{2\pi }},$$
  where
  5.4 $$\theta_{0}=\left|\sin^{-1}\left(\frac{r_{i}^{\left(\mathrm{br}\right)}}{a_{i}}\cos(\theta_{i}^{\left(\mathrm{br}\right)}-\beta_{\mathrm{br}}^{\left(i\right)})\right)\right|.$$
  Note that if either $a_{i}^{\left(1\right)}$ or $a_{i}^{\left(2\right)}$ is not equal to one of the radii *a*^(*l*)^, *l*=1,…,*N*_*r*_, we round it to the nearest one.
(v) Update the FSD by defining the vector *V*^(FSD)^_1_ containing the number of floes in the new array, i.e. after break-up has occurred, with each radius *a*^(*l*)^, *l*=1,…,*N*_*r*_.
(vi) Generate a random array of circular floes described by the FSD vector *V*^(FSD)^_1_. For this purpose, we use the random array generator devised by Montiel *et al.* [9] (see appendix B therein).
(v) Repeat steps (ii)–(vi) *N*_br_−1 times, where *N*_br_ is the number of break-up events considered for the simulation. At the end of each iteration *s*, we obtain an updated FSD defined by the vector *V*^(FSD)^_*s*_ for *s*=1,…,*N*_br_.

The break-up model described here should be seen as a new method to generate an FSD from repeated wave-induced floe break-up events. No attempt was made to replicate the fracture mechanism of ice floes in the MIZ with realistic shapes. From this perspective, the gross approximation of generating two circular floes from the break-up of a circular floe is acceptable, as we are only interested in the size of newly created floes as opposed to their shape. This approach is analogous to that of [19], in which floe size in the MIZ was measured by calculating the diameter of discs with the same area as that of the observed floes.

In step (v), we only include floes with radius *a*^(*l*)^≥*a*_crit_ in *V*^(FSD)^_*s*_ to generate the updated random array. Neglecting the influence of smaller floes on the break-up simulations improves the efficiency of the scattering computation in step (ii), noting that these small floes are still counted in the vector *V*^(FSD)^_*s*+1_.

It should be further noted that, although the break-up algorithm described here is intended to preserve the ice concentration (defined as the fraction of ice-covered ocean surface), rounding the radius of the two floes generated in step (iv) introduces a small change of concentration. However, simulations (not shown here) have revealed that these changes average out over a large number of break-up events, so the concentration actually remains quasi-constant.

## 6. Results

### (a) Single floe

We first conduct numerical experiments with the goal of understanding the FSD generated from the break-up of a single large ice floe under monochromatic and unidirectional wave forcing, as considered in §4b. We set *N*_f_=1 with *a*_max_=200 m. A sensitivity study (not shown here) demonstrated that choosing *a*_max_=500 m resulted in similar post-break-up FSDs, so the smaller radius was chosen for computational efficiency. We set *N*_*r*_=74, 84 and 100 unique floe radii between 5 m and *a*_max_ for *D*=1, 2 and 4 m, respectively, so that we have a 5 m resolution for *a*≥50, 60 and 80 m, respectively, and a 1 m resolution for smaller radii. Sensitivity tests (not shown here) indicated that the critical radius *a*_crit_, below which both scattering and break-up are consistently negligible, needs to be lowered from the values discussed in §4b for the cases *D*=4 m and *T*<10 s, probably because of the strong effect of multiple scattering. We, therefore, chose *a*_crit_=40, 40, 35, 30 and 30 m for *T*=5, 6, 7, 8 and 9 s.

The floe break-up algorithm described in §5 is then used to simulate the evolution of the FSD for *N*_br_=50 break-up events, which we find is generally sufficient to reach a steady state. The ice concentration is set to 50%, so that the random array of floes generated after each break-up event is enclosed in a square region with side length $\sqrt{2\pi }a_{\max}$.

The outputs of the break-up algorithm are the vectors *V*^(FSD)^_*s*_ for *s*=0,…,50, describing the FSD after *s* break-up events. These vectors can be interpreted as discrete functions of the floe radius variable *a* taking *N*_*r*_ values. The floe size probability density function (PDF), denoted by P(a), after *s* break-up events is defined as the linearly interpolated discrete function with values given in *V*^(FSD)^_*s*_ divided by the area under its curve. The PDFs are further averaged over 10 random realizations of the break-up simulation.

**Figure 4.**
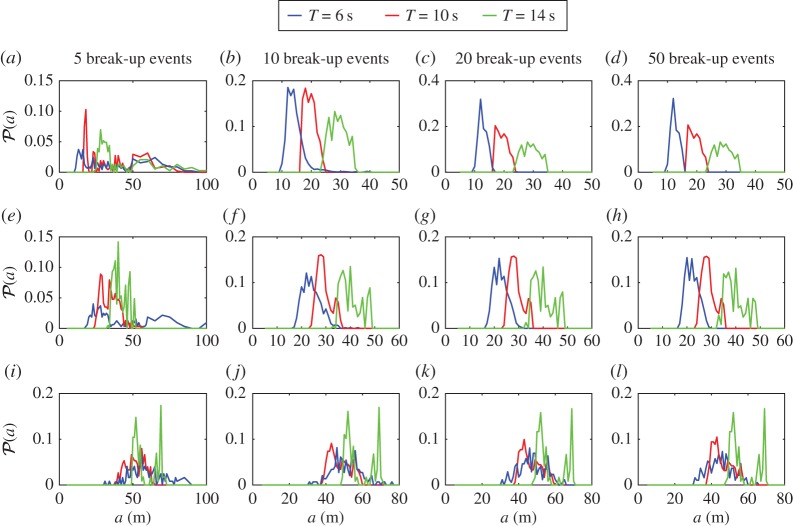
Evolution of the floe size PDF P(a) during the repeated break-up of a single floe with radius *a*_max_=200 m. Results are displayed for (*a*–*d*) *D*=1 m, (*e*–*h*) *D*=2 m and (*i*–*l*) *D*=4 m and wave periods *T*=6, 10 and 14 s (blue, red and green lines, respectively). The PDFs are shown after (*a*,*e*,*i*) *s*=5, (*b*,*f*,*j*) 10, (*c*,*g*,*k*) 20 and (*d*,*h*,*l*) 50 break-up events.

In figure 4, the floe size PDF is plotted after *s*=5, 10, 20 and 50 break-up events for the wave periods *T*=6 s (blue lines), 10 s (red lines) and 14 s (green lines) and the three ice thicknesses *D*=1 m (figure 4*a*–*d*), 2 m (figure 4*e*–*h*) and 4 m (figure 4*i*–*l*). The first striking feature is that the distributions obtained from the break-up simulations clearly cannot be identified as power-law distributions, as the number of floes decreases to zero for smaller and smaller radii. Instead, the distributions after 50 break-up events look either unimodal or multimodal, as the distributions contain one or multiple maxima. Closely spaced successive maxima, such as those seen for *T*=6 s and *D*=2 m around *a*=20 m, are likely to be an artefact of the floe radius sampling chosen for the simulations, so that they actually represent a single mode of the corresponding continuous distribution. We regard these oscillations as background noise. Accounting for this, we propose that all the PDFs are either unimodal or bimodal. Unimodality is observed for *D*=1 m at all wave periods and *D*=2 m for *T*=6 s, while bimodality seems to manifest itself in all other cases. The second mode for *D*=4 m at *T*=6 and 10 s is located at *a*≈55 m for both wave periods, although the magnitude of its peak is only slightly larger than the background noise, so caution is recommended in interpreting them as modes. It should be further noted that the unimodal distributions are all positively skewed, suggesting they may be the superposition of two closely spaced unimodal distributions. The bimodality may be explained by the fact that each floe with sufficient stress breaks into two floes only, so that the repeated break-up of the ice cover results in two dominant floe sizes that correspond approximately to the break-up of the smallest floe that can fracture for a given thickness and wave period. We do not attempt to analyse the bimodal property of the PDFs further.

For each parameter configuration considered in figure 4, we also observe a convergence of the PDFs with respect to the number of break-up events, as all distributions obtained for 20 events are almost identical to those obtained after 50 events, indicating that the break-up has ceased and a steady state has been reached after 20 events. The convergence seems to occur faster for longer waves and thicker floes, which is a consequence of fewer floe break-ups occurring for increasing values of these parameters.

**Figure 5.**
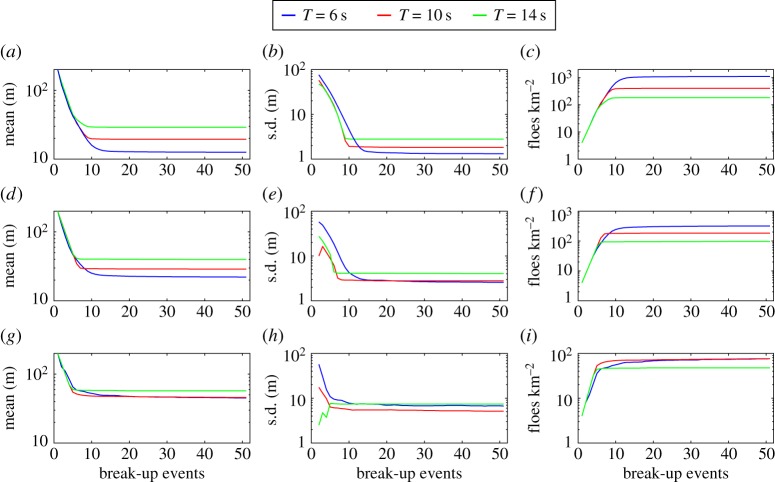
Evolution of (*a*,*d*,*g*) the mean and (*b*,*e*,*h*) standard deviation (s.d.) of the FSD, and (*c*,*f*,*i*) the number of floes per square kilometre, through the 50 break-up events. Each row of panels and line colour corresponds to a floe thickness and wave period, respectively, as defined in figure 4.

To understand better the convergence of the FSDs, figure 5 shows the evolution of the mean and standard deviation (s.d.) of the PDFs, and the number of floes per square kilometre through the 50 break-up events, for each wave period and thickness considered in figure 4. In the first few break-up events (i.e. up to *s*≈5), we observe that the statistics of the distribution change exponentially fast, corresponding to the regime in which all the floes in the array fracture, so that the number of floes doubles after each event. Interestingly, the mean floe size decreases independently of the wave period and floe thickness in this regime, while the standard deviation consistently remains higher at *T*=6 s than at the other periods for the three floe thicknesses considered, suggesting that shorter waves generate a broader FSD under intense wave-induced break-up as the ice cover fractures very quickly.

For *s*>5, the statistics of the FSD quickly reach a steady-state regime. The transition between the exponential break-up and steady-state regimes is very sharp for *T*=14 s, i.e. within five more break-up events, while it is longer for shorter waves, e.g. at *T*=6 s for which it can take more than 10–15 additional events. In particular, for a thickness *D*=4 m, a small number of floes are still breaking at the end of the simulation, i.e. *s*=50. Two processes are hypothesized to be responsible for the extended transition regime, namely (i) multiple wave scattering within the array of floes, which is expected to be strong for these parameters and to cause constructive interference that favours floe break-up, and (ii) mixing of the floes as a result of the randomization of the array of floes after each break-up event. (See step (vi) of the break-up algorithm described in §5.) Although this latter effect is an artefact of our break-up model which may amplify break-up, as large floes will ultimately end up in front of the array and then fracture, its influence on the final steady-state statistics of the FSD appears to be small for the single floe break-up simulations conducted in this section.

**Figure 6.**
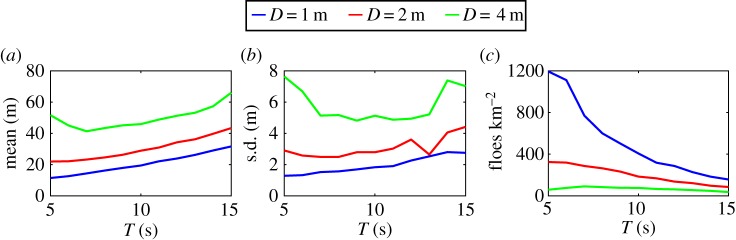
Steady-state statistics (i.e. after *s*=50 break-up events) plotted against wave period for floe thicknesses *D*=1, 2 and 4 m (blue, red and green lines, respectively). The statistics considered here are (*a*) the mean and (*b*) standard deviation of the FSD, and (*c*) the number of floes per square kilometre.

The dependence of the steady-state statistics (i.e. after *s*=50 break-up events) on the wave period and floe thickness is shown in figure 6. Mean and standard deviation are larger for increasing floe thicknesses, which is a result of thicker floes breaking less, as indicated in figure 6*c*, generating an FSD composed of a smaller number of larger floes. The larger standard deviation is explained by the stronger bimodality observed in the PDFs of the distribution for thicker floes, as can be seen in figure 4, because a larger separation of the two peaks results in a wider distribution.

For *D*=1 and 2 m, the mean and standard deviation smoothly vary with wave period with a general increasing trend for longer waves. This can also be explained by the fact that floe break-up diminishes for longer waves, which tends to enhance the formation of bimodal distributions. Note the minimum reached by the standard deviation at *T*=13 s for *D*=2 m. Inspection of the PDF obtained for this parameter configuration (not displayed here) shows that the bimodal shape of the distribution is not apparent, in contrast with *T*=12 and 14 s for which it clearly exists. We could not further explain this feature.

For *D*=4 m, the mean floe size reaches a minimum at *T*=7 s. It should be noted that this is smaller than *T*≈9 s for which the minimum of the critical radius *a*_crit_(*T*) discussed in figure 3 occurs for this thickness. This is likely to be a consequence of multiple wave scattering enhancing the stress field in the floes within the array at smaller wave periods, and break-up ensuing due to constructive interference. This further explains the need to take values of *a*_crit_ for the break-up simulations smaller than those computed in §4b, as discussed at the beginning of this section.

### (b) Array of floes

We now investigate how wave scattering by an array of floes influences the break-up process and associated evolution of its FSD. We consider the following initial configurations: (i) *N*_*s*_=10 slabs containing three floes each (i.e. *N*_f_=30) with thickness *D*=1 m and (ii) *N*_*s*_=20 slabs containing five floes each (i.e. *N*_f_=100) with thicknesses *D*=2 and 4 m. Subsequently, we refer to a slab of floes as a row. We chose a smaller array for *D*=1 m due to numerical constraints (thinner ice floes break up more, resulting in an increased computational cost required to solve the corresponding wave interaction problem with many floes). All the floes have initial radius *a*_max_=200 m and the ice concentration is 50%, so that arrays in configurations (i) and (ii) have approximate horizontal extents of 1.5×5 km and 2.5×10 km, respectively. The unique floe radii and critical radii *a*_crit_ for each thickness are the same as those chosen for the single floe break-up simulations in §6a. We perform the array break-up simulations for *N*_br_=50 break-up events. As a result of the significantly higher computational cost associated with the array break-up simulations compared with the single floe break-up simulations, no ensemble averaging was performed here, so all the results in this subsection are obtained from a single random realization for each wave period and floe thickness. Additional random realizations performed on a few selected cases showed remarkable consistency of the resulting FSD, suggesting very little variability exists in the stochastic process described here.

**Figure 7.**
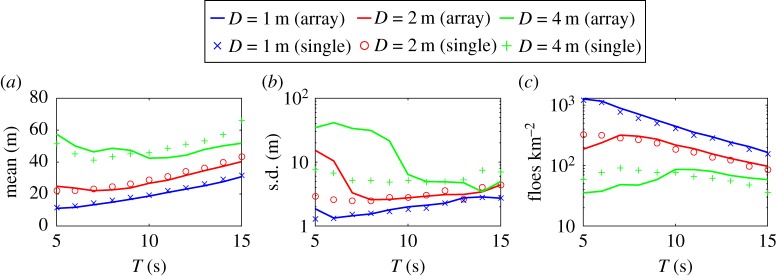
Same as figure 6 for the array simulations. The vertical scales in (*b*) and (*c*) differ from those of figure 6 and are logarithmic.

The mean, standard deviation and number of floes per square kilometre obtained after 50 break-up events for each wave period and floe thickness considered are plotted in figure 7, where they are compared with the steady-state statistics of the corresponding single floe break-up simulations. For *D*=1 m, the steady-state statistics obtained by breaking up the 10×3 array are very similar to those obtained from the break-up of a single floe, over the range of wave periods. It should be noted that the mean is consistently slightly smaller, while the number of floes is slightly larger, suggesting more break-up takes place for the array simulations, probably as a result of enhanced floe break-up due to constructive interference caused by multiple scattering. A similar observation can be made for *D*=2 m thick ice in the range of wave periods *T*≥7 s. For shorter waves, there is a clear deviation from the single floe break-up statistics, as the mean and standard deviation increase and the number of floes decreases as *T* decreases, which is the opposite trend to what happens for single floe break-up. This indicates that scattering is sufficiently dominant in this regime to prevent some floes from fracturing. The large increase in the standard deviation further suggests that the FSD spreads over a larger range of floe radii. Results obtained for *D*=4 m reinforce our explanations of the role of multiple scattering in break-up through the large array, with the observations of two regimes, i.e. *T*<10 s (short waves) and *T*≥10 s (long waves). In the short-wave regime, scattering generates sufficient wave energy attenuation to prevent floe break-up at some level of penetration in the ice-covered domain. In the long-wave regime, scattering enhances floe break-up due to constructive interference of the wave fields radiated by the freely floating individual ice floes.

**Figure 8.**
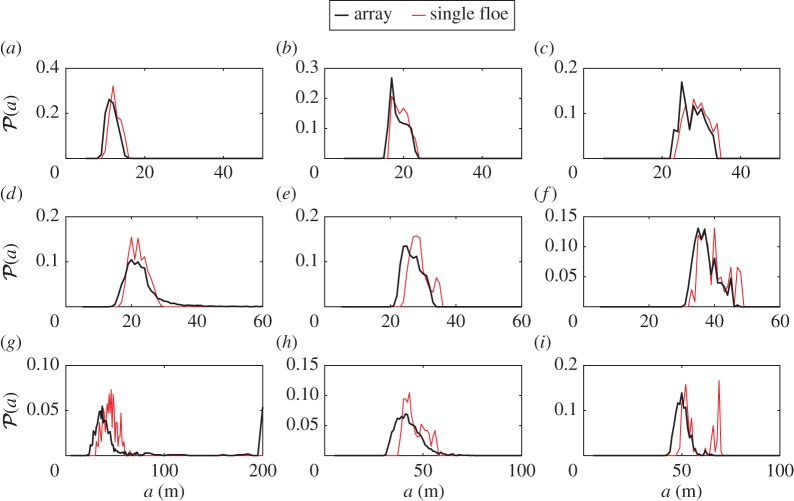
Comparisonof the steady-state PDFs P(a) obtained from the break-up of a large array (thick black line) and a single floe (thin red line) at wave periods (*a*,*d*,*g*) *T*=6 s, (*b*,*e*,*h*) *T*=10 s and (*c*,*f*,*i*) *T*=14 s. Results are displayed for (*a*–*c*) *D*=1 m, (*d*–*f*) *D*=2 m and (*g*–*i*) *D*=4 m. (Online version in colour.)

We seek more insight about the FSD obtained from the break-up of large arrays by plotting the PDFs of the distributions after 50 break-up events in figure 8. We can identify the two regimes discussed previously, i.e. enhanced break-up and reduced break-up, as a result of multiple wave scattering by a large array of floes. Based on our observations from figure 7, we associate enhanced break-up with the PDFs depicted in figure 7*a*,*b*,*c*,*e*,*f*,*i*, corresponding to cases for which scattering is not significant (i.e. long waves and/or thin ice). The PDFs show the presence of a larger number of small floes compared with the single floe break-up simulations, while the presence of larger floes decreases. Although this can be viewed as a shift of the PDF towards lower floe radii, the shape of the distribution also changes, particularly for thicknesses *D*=2 and 4 m. In these cases, the bimodality observed in the distributions obtained from the break-up of a single floe is not a persistent feature of the PDFs associated with the break-up of an array, as the second mode (i.e. the mode corresponding to larger floes) is damped or removed. For *D*=1 m, the PDFs are similar to those of the single floe break-up, being only slightly shifted towards smaller floe sizes.

The second regime, characterized by reduced break-up in the array, corresponds to the PDFs shown in figure 7*d*,*g*,*h*. The large spread of these distributions discussed above is clearly observed, with an increased presence of smaller floes (compared with the single floe break-up), as is the case in the long-wave/thin ice regime, as well as larger floes. Important wave scattering occurring in the front slabs of the array prevents wave energy from causing break-up deeper in the array. In the extreme case *D*=4 m and *T*=6 s depicted in figure 7*g*, we can see the presence of floes with radius *a*=200 m, which is the initial radius of all floes. Wave energy attenuation due to scattering is sufficiently strong in this case that some floes located deep enough in the array do not break.

**Figure 9.**
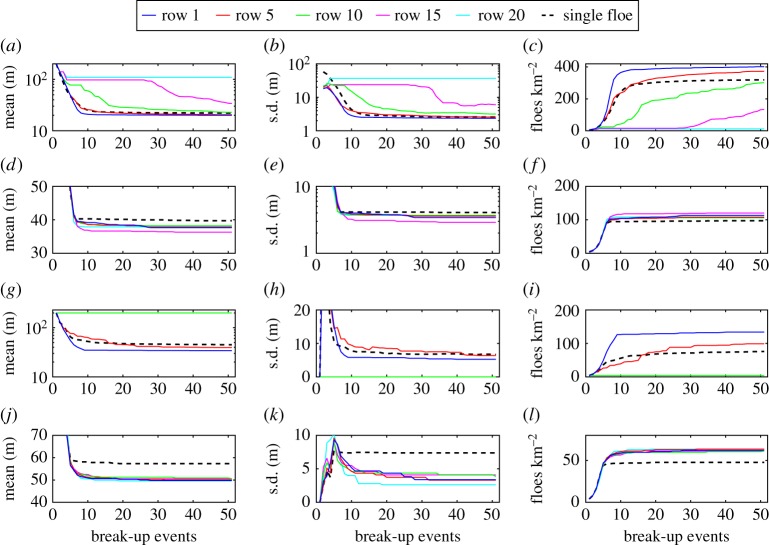
Evolution through 50 break-up events of (*a*,*d*,*g*,*j*) the mean, (*b*,*e*,*h*,*k*) standard deviation and (*c*,*f*,*i*,*l*) number of floes per square kilometre of the FSD in rows 1 (blue), 5 (red), 10 (green), 15 (magenta) and 20 (cyan) of the 20-row array used for simulations with ice thicknesses *D*=2 and 4 m. Results are shown for the four cases (*a*–*c*) *D*=2 m and *T*=6 s, (*d*–*f*) *D*=2 m and *T*=14 s, (*g*–*i*) *D*=4 m and *T*= 6 s, and (*j*–*l*) *D*=4 m and *T*=14 s. The evolution of the FSD statistics obtained for the single floe break-up are shown as black dashed lines for comparison.

To understand the role of scattering in the break-up of the array further, we focus our analysis on the four cases—(*D*,*T*)=(2 m,6 s), (2 m,14 s), (4 m,6 s) and (4 m,14 s)—depicted in figure 8*d*,*f*,*g*,*i*, respectively. Figure 9 shows the evolution of the mean, standard deviation and number of floes per square kilometre of the FSD in rows 1, 5, 10, 15 and 20 through the 50 break-up events. For the two long-wave cases (i.e. *T*=14 s) discussed here, we observe that the FSDs in all the rows converge to their steady state relatively uniformly. Interestingly, enhanced break-up compared with single floe break-up can be seen in all the rows. The level of break-up differs slightly between the rows, however. Inspection of the row dependence of the FSD steady-state statistics (not shown here) reveals an oscillatory behaviour with no clear trend in the case *D*=2 m, but with a clear peak in floe number around the 15th row, and a small upward trend in floe number for *D*=4 m, suggesting that break-up increases with the level of penetration in the array. The reader is reminded that the results presented in this section are based on a single random realization of the break-up simulations, so that an ensemble average may be necessary to resolve the small trends associated with the effect of penetration in the array on floe break-up.

For the two short-wave cases (*T*=6 s) considered in figure 8, we clearly observe the effect of scattering and associated wave energy attenuation in preventing floe break-up at some distance in the array. For *D*=2 m (figure 9*a*–*c*), the FSD in the first row converges quickly to a steady state, while that in row 5 also converges but at a slower rate as the steady state seems to be approximately reached towards the end of the simulation and with a significantly smaller number of floes than in row 1. This suggests that wave attenuation has an effect in reducing break-up in the first few rows. The evolution of the FSD deeper into the array is different, as we observe break-up taking place during the first four events but then suddenly stopping. Break-up then resumes in rows 10 and 15 after eight and 26 events, respectively, while it does not in row 20. It is hypothesized that large floes in these rows initially fracture, as they do not require much energy to reach the critical break-up stress, as suggested in figure 2. After a few break-up events, the floes become too small to break under a wave field strongly attenuated by the front rows. As break-up in these front rows continues, however, wave attenuation due to scattering by smaller and smaller floes also decreases, so that more wave energy propagates further into the array with the result that floes are gradually broken up deeper and deeper in the ice field. In other words, we have shown in our model that wave-induced break-up has the capacity to reduce the structural integrity of the MIZ, enabling waves to travel further and cause break-up there, further weakening the ice cover. This positive feedback process is often used as a motivational concept for observational studies on wave–sea ice interactions. Note that a steady state is not reached after 50 break-up events in this simulation, so it is possible that break-up starts to resume in row 20 after the rows in front have experienced sufficient break-up.

In the case *D*=4 m (figure 9*g*–*i*), break-up in rows 1 and 5 behaves similarly to that for *D*=2 m, but rows 10 onwards do not experience break-up during the 50 events simulated here. Because thicker ice tends to increase the degree of wave attenuation, this is a plausible outcome of the simulation. Inspection of the evolution of the FSD in rows 6, 7, 8 and 9 (not shown here) indicates that break-up is ongoing after 50 events, so that rows 10 onwards may gradually start breaking after a larger number of break-up events.

### (c) Wave spectrum

We now consider the break-up of the arrays used in the previous section under a unidirectional wave spectrum, in part acknowledging that the FSD observed in ice-covered oceans is the result of break-up by a sea state composed of a range of frequency and directional components. We do not seek to reproduce the break-up response to an observed random sea state in the present model, but instead we conduct a controlled numerical experiment to assess qualitatively how the FSD induced by an ad hoc wave spectrum differs from that obtained under monochromatic wave forcing. For this purpose, we sample wave components from a two-parameter Bretschneider spectrum defined by the spectral density function

6.1 $$S(T)=\frac{5}{32\pi }T_{p}H_{s}^{2}{\left(\frac{T}{T_{p}}\right)}^{5}\;e^{-(5/4){\left(T/T_{p}\right)}^{4}},$$

where *T*_p_ is the peak wave period and *H*_s_ is the significant wave height [49]. Sampling the period spectrum at integer wave periods between 5 and 15 s, the amplitude of the ambient field at each centre period of 1 s bandwidth is given by

6.2 $$A^{\mathrm{am}}(\tau )=\sqrt{2S(T)}\delta (\tau ).$$

At this resolution, the incident wave amplitude is close to 1 m for each sampled period, being slightly larger around the peak period and slightly smaller away from it, and hence we expect break-up to occur for all periods, which is our intent in using this ad hoc model. Choosing a finer resolution would reduce the amplitude of each component and may not cause sufficient break-up to analyse the FSD and compare it with that obtained under monochromatic forcing.

For our break-up simulations, we select one wave period *T*∈[5,15] randomly at each event and compute the break-up induced by a unidirectional plane wave of period *T* with amplitude given by (6.2). An ensemble of 10 random realizations of the break-up simulation is computed for each ice thickness. Although this approach is potentially different from break-up by a wave field composed of multiple frequencies, it is conjectured that the randomization of both wave period and array in conjunction with ensemble averaging provides a legitimate approximation. We set the parameters of the spectrum to *T*_p_=10 s and *H*_s_=2 m, which corresponds to a typical swell observed in the Southern Ocean [5].

**Figure 10.**
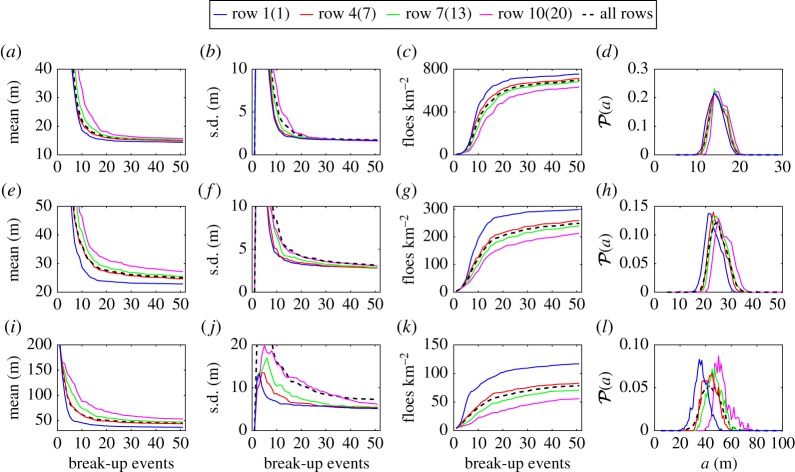
Evolution of (*a*,*e*,*i*) the mean, (*b*,*f*,*j*) the standard deviation and (*c*,*g*,*k*) the number of floes per square kilometre of the FSD, and (*d*,*h*,*l*) PDFs of the FSD after 50 break-up events. Results are shown for the floe thicknesses (*a*–*d*) *D*=1 m, (*e*–*h*) *D*=2 m and (*i*–*l*) *D*=4 m, and rows 1(1), 4(7), 7(13) and 10(20) in the arrays of floes with *D*=1 m (2 and 4 m) as solid blue, red, green and magenta lines, respectively. We also include results for the entire array (black dashed line) for comparison.

 Figure 10 shows the evolution of the mean, standard deviation and number of floes per square kilometre of the FSD for different rows of the array and for the entire array (figure 10*a*–*c*,*e*–*g*,*i*–*k*). We observe a clear convergence trend towards a steady state for all rows of the array and for each thickness. The front rows consistently converge faster than the back ones and to a lower mean and a higher number of floes, so that the degree of break-up decreases with the level of penetration in the array. Interestingly, the evolution of the mean and number of floes of the FSD for the entire array almost coincides with those associated with the middle row of the array. This suggests a simple linear dependence of the FSD statistics with respect to the row number, which will be demonstrated later. The standard deviation of the FSD for the entire array is consistently at least as large as that of the last row, which in turn is the largest of all the rows. This reflects the larger spread of floe sizes on a large scale (entire array) compared with the local scale (each row).

We further display the PDF of the FSD for all cases discussed above in figure 10*d*,*h*,*l*. Observe first that the distributions look nearly normal. This is probably the result of effectively averaging over the wave periods. The bimodality seen in previous distributions does not completely disappear, however, as a shoulder-type feature can be seen on the right of the peak (i.e. for large radii), particularly for *D*=1 and 2 m. This suggests the existence of a second mode for large radii but with a small peak.

**Figure 11.**
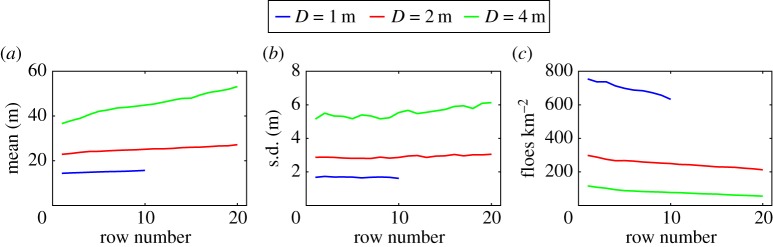
(*a*) Mean, (*b*) standard deviation and (*c*) number of floes per square kilometre after 50 break-up events as a function of row number.

The row dependence of the mean, standard deviation and number of floes after 50 break-up events is analysed further in figure 11. As indicated above, we observe a linear increase in the mean of the FSD and a linear decrease in the number of floes with respect to row number. This allows us to estimate the rate of change of mean floe size with respect to penetration in the array, which is important for large-scale modelling studies of the MIZ. Fitting a straight line through the curves generated and extracting the slope, we find that the mean floe radius increases at a rate of 0.27, 0.39 and 1.5 m per kilometre of penetration for *D*=1, 2 and 4 m, respectively. This notwithstanding, it is unclear how the limited size of the array affects these estimates.

## 7. Conclusion

A new model of ice floe break-up under ocean wave forcing in the MIZ has been developed. It combines the time-harmonic multiple scattering theory for a finite array of floating elastic discs proposed by [9] with a parametrization of flexural failure causing an ice floe to fracture into two floes, provided that the stress field satisfies a particular break-up criterion, the so-called MC criterion. We derived a quantity, referred to as the MC stress, that uniquely defines the level of stress at each point of the surface of an ice floe, allowing us to test simply if break-up is expected to take place at any point. A numerical experiment was then conducted to analyse the MC stress experienced by a single ice floe under a unit amplitude unidirectional wave forcing and determine the regime in which we expect floe break-up. It was found that

(i) a minimum floe diameter exists for each thickness below which break-up cannot occur, and
(ii) this critical diameter depends on the wave period in a way that it reaches a minimum at a resonant wave period, for which the floe diameter is approximately equal to the open water wavelength and half the ice-covered wavelength.

A closed-loop feedback algorithm has been proposed to model the evolution of the FSD in the MIZ under a sustained wave event. Each loop consists of (i) computing the MC stress in all the floes, (ii) breaking up each floe satisfying the MC criterion, and (iii) generating a new array of floes from the updated FSD, which is then used as the geometry of the wave interaction problem in the next loop. We conducted a number of numerical experiments to determine the evolution of the FSD towards a steady state through 50 break-up events (i.e. loops), for different wave and ice configurations. We simulated the break-up of (i) a single large floe for a monochromatic unit-amplitude plane wave forcing, (ii) an array of large floes for the same monochromatic forcing, and (iii) an array of large floes for a Bretschneider spectrum forcing. Key findings are summarized below.

(i) Break-up of a single large floe causes the emergence of a bimodal FSD for most wave and ice parameters considered. Larger values of wave period and ice thickness correspond to FSDs with larger floe sizes and more separated peaks of the associated bimodal distribution. The convergence of the FSD towards its steady state under repeated break-up events is very quick for long waves and slower for short waves. Increasing values of ice thickness also tends to decrease the rate of convergence of the FSD, suggesting that multiple wave scattering within the array of broken floes enhances break-up and therefore influences the steady-state FSD.
(ii) Break-up of an array of large floes under monochromatic forcing provides additional insight into the effect of multiple scattering on the FSD. First, the bimodality of the FSD observed for the single floe break-up simulations is either damped or removed, as the population of large floes associated with the second mode is consistently redistributed to smaller floes. Second, results of our simulations indicate that two scattering regimes exist, i.e. long waves/thin ice and short waves/thick ice. In the former regime, wave-induced ice break-up is enhanced compared with the single floe break-up, which is likely to be a consequence of constructive interference of wave fields radiated by the individual floes. In the second regime, multiple scattering causes sufficient wave energy attenuation through the array to prevent some ice floes from fracturing, resulting in broader FSDs compared with those obtained from the repeated break-up of a single floe.
(iii) Investigation of the evolution of the FSD at different levels of penetration in the array indicates that enhanced floe break-up in the long-wave regime occurs throughout the array with a small upward trend with distance from the ice edge. In the short-wave regime, we observe the positive feedback between wave-induced ice break-up and ice-induced wave attenuation. Break-up originally only takes place in the front rows as waves are attenuated by scattering deeper in the array. After sufficient break-up, waves are less attenuated, carry energy at a higher level of penetration and cause break-up there. The overall effect is the observation of a break-up front marching forward in the MIZ as the structural integrity of the ice cover reduces under wave-induced break-up.
(iv) Break-up of an array under a Bretschneider spectrum forcing generates near-normal FSDs for all ice thicknesses, which is likely to be a consequence of averaging with respect to the wave period. As opposed to the simulations with monochromatic forcing, break-up decreases with the level of penetration in the array, such that the mean floe size increases linearly with distance from the ice edge. The rate of floe size increase is larger for thicker ice.

An important outcome of our investigation is that no power-law FSD was generated from the simulations. Our model consistently predicts that the number of floes decreases to zero for smaller floe sizes in an MIZ with uniform ice thickness. This results from the fact that small floes are less prone to elastic deformations than large floes, i.e. they behave similarly to a rigid body. Analysis of the data generated in §4b shows that the wave energy required to cause break-up in small floes increases exponentially fast as floe size decreases below a critical size, so that flexural failure by ocean waves is unlikely to be responsible for the observed increasing number of small floes in the MIZ [19], acknowledging that small floes with size *O*(1 m) cannot be resolved accurately by current observation techniques, so that it is unclear whether the power law extends to very small scales. In any case, our analysis suggests that wave-induced floe break-up alone does not create or preserve the observed power-law FSD or the apparent increase in small floe numbers, but may partially contribute to it. Other processes acting on longer time and/or length scales (e.g. thermodynamics, internal stress or collisions) must be considered to explain this feature of the FSD. Although recent modelling work has shown the emergence of a power-law FSD [27,28,50], it is still unclear how much each process contributes to the observed result. It should also be noted that we attempted to fit a power-law curve in the large floe regime of the FSDs obtained from our simulations, i.e. for radii larger than the peak radius, but the limited extent of this regime (i.e. spanning less than one order of magnitude in floe radius) did not allow us to obtain statistically significant results.

Although our findings provide much theoretical understanding of the wave-induced ice break-up process in the MIZ, the underlying model was constructed based on a number of simplifying assumptions, which may influence certain results. Specifically, the FSD resulting from the break-up of a more heterogeneous ice cover, i.e. governed by a floe shape and ice thickness distribution and possibly different ice types, under a realistic multidirectional random sea state, may spread the FSD over a wider range of floe sizes to the point where a power law could be fitted to a portion of the curve. In addition, the validity of our break-up model, which involves breaking a circular floe into two circular floes and time-harmonic wave forcing, is unclear and requires further investigation. It would be difficult to relax these assumptions in the context of the three-dimensional wave-scattering model considered here. We may envisage a simpler two-dimensional model, however, in which the one-dimensional ice cover is initialized as a semi-infinite beam and forced by a transient incident wave generated from a frequency spectrum. More theoretical investigation into this physical process is needed to be able to provide large-scale ice–ocean wave forecasting models with a quantitative parametrization of the two-way coupling between ocean waves and sea ice. Monitoring the evolution of the FSD in an area of ice-covered ocean during a wave break-up event either in the field or in a controlled laboratory setting, with the ability to resolve small floes accurately, would be needed to provide a clearer picture of the complicated processes investigated here.

## Data accessibility

The Matlab codes and data files created for the numerical investigation reported here can be accessed through http://www.maths.otago.ac.nz/files/icebreakup/Data_breakup.zip.
